# The neonatal marmoset monkey ovary is very primitive exhibiting many oogonia

**DOI:** 10.1530/REP-14-0068

**Published:** 2014-08

**Authors:** B Fereydouni, C Drummer, N Aeckerle, S Schlatt, R Behr

**Affiliations:** Stem Cell Biology Unit German Primate Center – Leibniz Institute for Primate Research Kellnerweg 4, Göttingen, 37077 Germany; 1 Centre of Reproductive Medicine and Andrology University of Münster Domagkstraße 11, Münster, 48149 Germany

## Abstract

Oogonia are characterized by diploidy and mitotic proliferation. Human and mouse oogonia express several factors such as OCT4, which are characteristic of pluripotent cells. In human, almost all oogonia enter meiosis between weeks 9 and 22 of prenatal development or undergo mitotic arrest and subsequent elimination from the ovary. As a consequence, neonatal human ovaries generally lack oogonia. The same was found in neonatal ovaries of the rhesus monkey, a representative of the old world monkeys (Catarrhini). By contrast, proliferating oogonia were found in adult prosimians (now called Strepsirrhini), which is a group of ‘lower’ primates. The common marmoset monkey (*Callithrix jacchus*) belongs to the new world monkeys (Platyrrhini) and is increasingly used in reproductive biology and stem cell research. However, ovarian development in the marmoset monkey has not been widely investigated. Herein, we show that the neonatal marmoset ovary has an extremely immature histological appearance compared with the human ovary. It contains numerous oogonia expressing the pluripotency factors OCT4A, SALL4, and LIN28A (LIN28). The pluripotency factor-positive germ cells also express the proliferation marker MKI67 (Ki-67), which has previously been shown in the human ovary to be restricted to premeiotic germ cells. Together, the data demonstrate the primitiveness of the neonatal marmoset ovary compared with human. This study may introduce the marmoset monkey as a non-human primate model to experimentally study the aspects of primate primitive gonad development, follicle assembly, and germ cell biology *in vivo*.

## Introduction

Primates can be subdivided into two large groups: Strepsirrhini (the former group of prosimians excluding tarsiers) and Haplorhini (the former group of simians plus tarsiers) (Finstermeier *et al*. 2013). The old term *simian* has been replaced by Anthropoidea (i.e. Haplorhini without tarsiers). Anthropoidea include Platyrrhini (new world monkeys) and Catarrhini (old world monkeys, and Hominoidea, which include humans, great apes, and gibbons) (Perelman *et al*. 2011, Finstermeier *et al*. 2013). The Strepsirrhini is a group of primates with characteristics often considered rather primitive compared with Anthropoidea. Old world monkeys are native to Asia and Africa, while the new world monkeys are native to America. In human, a representative of the Catarrhini, the adult ovary provides the gametes from a fixed pool of germ cells, which was established during the fetal phase of ovary development (Hartshorne *et al*. 2009), and there is evidence that there is no germ cell proliferation in the normal postnatal human (Stoop *et al*. 2005, Liu *et al*. 2007, Byskov *et al*. 2011) and old world monkey ovaries (Yuan *et al*. 2013). By sharp contrast, studies carried out more than 40 years ago show the presence of mitotically proliferating germ cells in the ovary of even adult Strepsirrhini (Ioannou 1967, Butler & Juma 1970). In the human ovary, the first oogonia enter meiosis during embryonic week 9 (Bendsen *et al*. 2006). During weeks 10–12 of ovarian development, oogonia constitute 50–60% of all ovarian germ cells (Maheshwari & Fowler 2008). During weeks 12–18, the number of oocytes increases reflecting the entry into meiosis of most germ cells (Maheshwari & Fowler 2008). The vast majority of oogonia had entered meiosis by the end of the second trimester (Stoop *et al*. 2005, Bendsen *et al*. 2006), so that the late fetal human ovary already contains almost only proliferation-arrested germ cells. Recently, Byskov *et al*. (2011) have reported that oogonia are very rarely present in peri- and postnatal human ovaries, and in previous histological analyses no oogonia were found in neonatal human and macaque ovaries (van Wagenen & Simpson 1973). Oogonia and oocytes can be cytologically distinguished based on their contour in the histological sections and their nuclear structure (Maheshwari & Fowler 2008). Particularly, synaptonemal complex formation is a major hallmark of meiotic prophase (Maheshwari & Fowler 2008). At the molecular level, it has been shown in the human fetus that specifically premeiotic germ cells (primordial germ cells and oogonia and gonocytes respectively) maintain the expression of some pluripotency markers whose expression is otherwise restricted to the inner cell mass of preimplantation embryos and to *ex vivo* cultured pluripotent stem cells (Kerr *et al*. 2008, Perrett *et al*. 2008, Childs *et al*. 2012).

In this study, we used the common marmoset monkey (*Callithrix jacchus*) as a representative of the new world monkeys (Platyrrhini) to study its germ cell population in the immature ovary. The body size of only ∼25 cm and a weight of 300–450 g make marmoset monkeys an easy-to-handle animal model yet representing primate physiology and biology. High reproductive success and absence of reproductive seasonality are key characteristics making marmoset monkeys a very valuable model to study non-human primate (NHP) reproductive physiology and stem cells (Harlow *et al*. 1983, Mansfield 2003, Sasaki *et al*. 2005, 2009, Chandolia *et al*. 2006, Luetjens *et al*. 2008, Mitchell *et al*. 2008, McKinnell *et al*. 2009, 2013, Albert *et al*. 2010, Beindorff & Einspanier 2010, von Schonfeldt *et al*. 2011, Aeckerle *et al*. 2012, Hanazawa *et al*. 2012, Lin *et al*. 2012, Tomioka *et al*. 2012, Parte *et al*. 2013). However, research on marmoset gonadal development has only been focused, to our knowledge, on the male (Li *et al*. 2005, Chandolia *et al*. 2006, Mitchell *et al*. 2008, McKinnell *et al*. 2009, 2013, Albert *et al*. 2010, Aeckerle *et al*. 2012, Eildermann *et al*. 2012, Lin *et al*. 2012). Consequently, almost nothing is known about the characteristics of the neonatal marmoset monkey ovary.

In this study, we used five protein markers, namely OCT4A (also called POU5F1), SALL4, LIN28A (LIN28), VASA (also called DDX4), and MKI67 (Ki-67), to study the germ cell population in the marmoset monkey ovary. The *OCT4A*-isoform of the *OCT4* gene is germ line specific and is one of the most specific and indicative markers of pluripotency (Scholer *et al*. 1990, Liedtke *et al*. 2008, Wang & Dai 2010). OCT4 is important for mouse primordial germ cell survival (Kehler *et al*. 2004) and was also used to study human germ cell development (Stoop *et al*. 2005, Perrett *et al*. 2008, Byskov *et al*. 2011, Childs *et al*. 2012, Mamsen *et al*. 2012). The transcription factor SALL4 is essential for pluripotency of mouse ES cells (Elling *et al*. 2006). Mouse spermatogonia also critically depend on SALL4 function (Hobbs *et al*. 2012). We demonstrated that SALL4 protein expression is restricted to premeiotic germ cells in the human and NHP testes including the common marmoset monkey testis (Eildermann *et al*. 2012). The RNA-binding pluripotency-associated protein LIN28A is expressed in marmoset monkey ES cells and in human and monkey male premeiotic germ cells (Aeckerle *et al*. 2012, Vogt *et al*. 2012). In the human ovary, LIN28A has been shown to be a specific marker of oogonia (Childs *et al*. 2012). The functional importance of LIN28A for the formation of mouse germ cells has also been demonstrated (West *et al*. 2009). VASA is a germ-line-specific RNA helicase enzyme that is expressed in most intragonadal stages of male and female human germ cell development from premeiotic up to the postmeiotic stages (Castrillon *et al*. 2000, Anderson *et al*. 2007). However, first trimester human gonocytes and oogonia generally lacked VASA (Anderson *et al*. 2007).

MKI67 protein is a nuclear protein, which is present during the G1, S, G2, and M phases of the cell cycle but absent in resting cells in G0 phase. Hence, MKI67 specifically marks the proliferative cell population in a given tissue and represents one of the most reliable proliferation markers (Scholzen & Gerdes 2000). In human ovarian germ cells, MKI67 is strongly expressed by oogonia, while oocytes were generally negative for MKI67 (Stoop *et al*. 2005). Only occasionally, few oocytes engaged in primordial follicles showed some MKI67 signals, which were strictly confined to the nucleolus. Therefore, in human, nuclear MKI67-positive ovarian germ cells represent oogonia.

The aims of this study were i) to describe marmoset monkey ovarian histology at birth and ii) to characterize the germ cell population in the neonatal ovary of the common marmoset monkey with regard to the presence of oogonia. We show that the neonatal marmoset ovary is, compared with the human ovary, extremely primitive at birth, as it contains numerous premeiotic germ cells. Therefore, the postnatal marmoset ovary may represent a useful NHP model to experimentally study phases of female gonadogenesis, which in humans occur during intrauterine embryonic/fetal development, which is experimentally inaccessible.

## Materials and methods

### Animals

Common marmoset monkeys (*C. jacchus*) were studied. Monkeys were housed in a self-sustaining colony at the German Primate Center (Deutsches Primatenzentrum; DPZ) according to standard housing for marmoset monkeys. The institutional guidelines of the DPZ for the care and use of marmoset monkeys were strictly followed. According to applicable law, no separate license is necessary to harvest organs after killing of the animals. Neonatal female common marmoset monkeys (aged 1–5 days) were obtained from the breeding colony of the German Primate Center (Göttingen, Germany). The marmosets were housed as breeding pairs. In captivity, they often give birth to triplets or even quadruplets. However, the mother is usually able to feed and rear only two neonates, which is the normal litter size in free-living marmosets. Therefore, the female neonates from triplet or quadruplet births were used to collect organs for this study. The marmosets were housed in a temperature-controlled (25±1 °C) and humidity-controlled (65±5%) facility. The marmosets were maintained in 12 h light:12 h darkness cycle. The animals were fed *ad libitum* with a pelleted marmoset diet. In addition, 20 g mash per animal were served in the morning and 30 g cleanly cut fruits or vegetables mixed with noodles or rice were supplied in the afternoon. Drinking water was also available *ad libitum*.

### Numbers of animals

Six neonatal marmoset ovaries from four different animals in the age range of postnatal days 1–5, one 1-year-old marmoset ovary, four adult ovaries from three different animals, and one fetal ovary were used in this study.

### Retrieval of tissues

Neonatal marmoset monkeys were obtained from the DPZ breeding colonies and anaesthetized with pentobarbital (Narcoren; 0.05 ml i.m.) and then killed by an intracardial injection of 0.5 ml pentobarbital. Neonatal marmosets were selected from triplet births based on the changes in body weight of the neonates. The ones that had the lowest birth weight or the ones that lost most weight during the first 1–5 postnatal days due to the inability of the mother to nourish three offspring were selected for killing. All animals were killed before a lack of nourishment caused suffering of the animals. The 1-year-old marmoset ovary was obtained from the DPZ pathology unit. The animal was killed due to a reason unrelated to reproductive functions and the ovary was made available to this study. Ovaries from adult females were available from the histological archive of the DPZ. The fetal ovary (calculated gestational day 90; total period of gestation is ∼144 days in marmoset monkeys; Chambers & Hearn (1979)) was obtained from a fetus from the breeding colony of the Centre of Reproductive Medicine and Andrology (CeRA) of the University Hospital Münster. The fetus was recovered from the uterus of a pregnant female after killing of the female (license number 8.87-50.10.46.09.018). The age of the fetus was calculated as approximately gestational day 90. This was based on the breeding history of the female and on the development of the fetus in comparison with other fetuses obtained from timed pregnancies in another study. The normal period of gestation of marmosets is 143 days.

### Histology and immunohistochemistry

Ovaries and the fetus were immediately fixed in Bouin's solution, further processed according to routine histological techniques, and eventually embedded in paraffin for immunohistochemical analysis. Serial sectioning (5 μm sections) was performed and the sections were placed on adherent slides. Paraffin sections were de-waxed and rehydrated. Antigen retrieval was carried out with the 10 mM Na-Citrate buffer at pH 6.0 for 10 min in high-power microwave. Paraffin sections were washed for 5 min with 0.05 M Tris buffer. Then blocking of peroxidase was done for 15 min with the Peroxidase block (Dako, Hamburg, Germany). After washing again with a washing buffer, immunostainings were done with OCT4A (#2890S, Cell Signaling Technology, Frankfurt am Main, Germany) (1:100), LIN28A (#3978S, Cell Signaling Technology) (1:100–1:200), SALL4 (#ab57577, Abcam, Cambridge, UK) (1:200), VASA (DDX4; #AF2030, R&D Systems, Wiesbaden-Nordenstadt, Germany) (1:100), and MKI67 (#9027S, Cell Signaling Technology) (1:300). The antibodies were also used in previous studies (Aeckerle *et al*. 2012, 2013, Eildermann *et al*. 2012) and were found to be valid for the detection of the respective marmoset monkey proteins. Primary antibodies were diluted with a washing buffer plus 5% BSA. Sections were incubated with the primary antibodies at 4 °C overnight. The primary antibodies were detected using the DAKO LSAB+ system- HRP kit (#K0679). Sections were visualized with the chromogen DAB that showed brown signals. Mayer's hematoxylin staining was used as a counterstain. Negative controls were performed by omitting primary antibodies and using corresponding non-specific immunoglobulin controls instead of the specific primary antibody. Double-staining was performed using the DAKO EnVision G|2 Double stain System and Rabbit/Mouse (DAB+/Permanent Red) kit (ordering number K5361) according to the manufacturer's protocol. The photos were captured using a ZEISS Axiophot microscope equipped with a Nuance camera, which is a multispectral camera enabling imaging of multiple markers on tissue sections also in bright-field microscopy and even when the signals co-localize.

### RT-qPCR

RT-qPCRs for a pluripotency marker (*OCT4A, POU5F1*), pluripotency/germ cell markers (*LIN28A, SALL4*), and a germ cell marker (*VASA; DDX4*) were performed (*n*=3 independent PCR runs with all samples). Primers are listed in Table 1. Total RNAs from newborn ovaries (*n*=5), from different passages of an embryonic stem cell line (*n*=4; ES cell line Cjes001), and from different passages of fibroblasts derived from one primary culture (*n*=4) of the common marmoset monkey were extracted using the QIAGEN RNeasy Mini Kit according to the manufacturer's instructions. RNA samples were treated with DNase I to digest contaminating DNA. cDNA was produced from 1 μg RNA using the QIAGEN Omniscript reverse transcriptase kit. The Master mix for every reaction consisted of 2 μl 10× buffer, 2 μl dNTPs, 2 μl Oligo-dT, and 1 μl reverse transcriptase in a final volume of 20 μl. RT was performed at 37 °C for 1.5 h in the thermocycler (Biometra T3000, Goettingen, Germany). The resulting cDNA was diluted 1:5 and was then used as a template for each 20 μl PCR with the power SYBER Green PCR Master Mix (#4368706, Applied Biosystem). Appropriate primer concentrations for each primer pair (see Table 1) were tested to yield a distinct, single amplicon, which was visualized by 2% agarose gel electrophoresis. Identity of the amplicon was confirmed by DNA sequencing. The qPCR program consisted of an initial step of denaturation (10 min at 95 °C) followed by 40 cycles of denaturation (15 s at 95 °C) and annealing/elongation (1 min at 60 °C). As qPCR controls we included a no-template control, which contained all real-time PCR components except the template, a −RT (Reverse Transcriptase) control and positive controls (ES cell RNA) to test for the presence of PCR inhibitors. Each sample was assayed in triplicate and normalized to glyceraldehyde-3-phosphate dehydrogenase (*GAPDH*) expression. The expression level of *GAPDH* was stable between the samples. Relative quantification was based on the method used (Livak & Schmittgen 2001). Statistical analysis (unpaired *t*-test) was performed using the Graphpad Prism software (http://www.graphpad.com/quickcalcs/ttest1.cfm). *P*<0.05 was considered as statistically significant.

## Results

### Histology of the neonatal marmoset ovary

**Figure 1:**
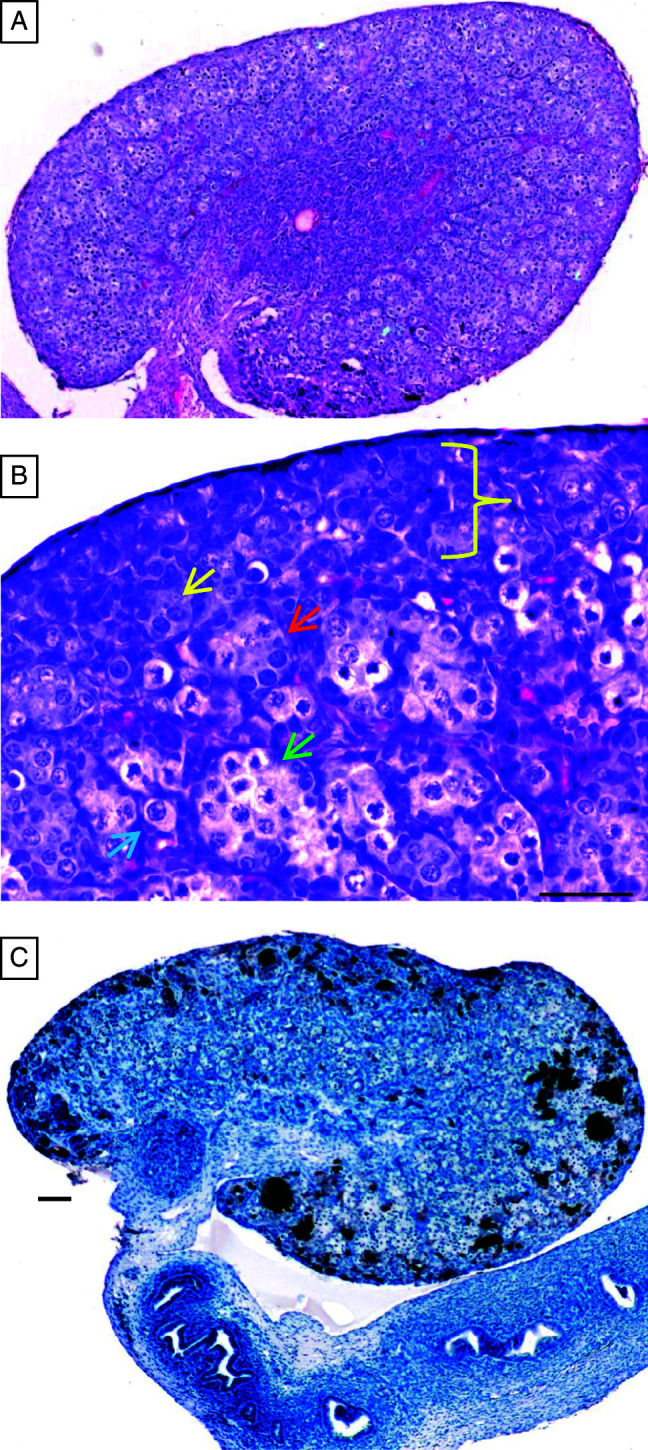
Histology of the neonatal marmoset monkey ovary. (A) An overview of the whole cross-section through a neonatal ovary. The central medulla region and the peripheral cortical region can be easily recognized. The whole ovary is covered by the ovarian surface epithelium (OSE). Between the outer zone of the cortex and OSE, there is a histological layer called indifferent cortical zone (ICZ) of the neonatal marmoset ovary (see also B). At the bottom, the hilum/mesovary can be observed. (B) A higher magnification of the peripheral zones of the ovary. The dark line covering the tissue represents the flat OSE. The bottom part shows the classical cortical zone characterized by cysts of germ cells and few primordial follicles. The ICZ is indicated by the yellow bracket. A *Tunica albuginea*, a characteristic sub-OSE layer of the adult ovary, is not yet established. Yellow arrow, nest of oogonia; red arrow, nest containing some oogonia and some oocytes. Note the different nuclear structures of the germ cells. Green arrow, nest of oocytes; blue arrow, primordial follicle. (C) An overview of the distribution of premeiotic germ cells in the neonatal ovary. The brown stain indicates the presence of a specific oogonial marker (LIN28A). Stained cells are present in the cortical zone, in the ICZ, and in the OSE either as large clusters, smaller groups of cells, or as isolated cells (for further details, see Fig. 3). The different somatic cells of the ovary as well as the different cell types of the oviduct (bottom part of C) are not stained. The scale bars represent 50 μm.

Figure 1A is an overview over a whole cross-sectioned neonatal marmoset monkey ovary. The surface of the neonatal ovary is regular and there is no lobular structure. The entire ovary is covered by the ovarian surface epithelium (OSE). Positioned directly underneath the OSE there is a thin layer of cells in which the different cell types are histologically hardly distinguishable. It contains primitive germ and somatic cells and is shown at a higher magnification in Fig. 1B. This zone is termed indifferent cortical zone (ICZ) of the neonatal marmoset ovary. A typical *Tunica albuginea* as a connective tissue layer underneath the OSE is not yet established (compare with histology of 1-year-old marmoset ovary). The next layer and major compartment of the neonatal marmoset ovary is the immature cortex, where the germ cells are still organized in clusters or nests of cells. These germ cell aggregations are ‘belted’ by somatic cells. The central part of the marmoset ovary is constituted by the medulla. The mesovary can be seen in the lower left part of Fig. 1A.

At the border between the ICZ and the cortex, there are germ cell nests containing both, germ cells with an oogonia phenotype and germ cells with an oocyte (meiotic) phenotype (Fig. 1B). Moreover, there are numerous small nests of cells containing apparently only oogonia. The deeper areas of the cortex contain predominantly cell nests with meiotic germ cells. Few primordial follicles are also present. In order to obtain an overview over the presence and distribution of premeiotic germ cells in the neonatal marmoset ovary, we used LIN28A immunohistochemistry (Fig. 1C). The overview of a LIN28A-stained ovary indicates an abundance of premeiotic germ cells in the neonatal marmoset ovary. Two characteristics are evident: i) there are huge clusters or nests of oogonia as well as scattered oogonia and ii) oogonia are present not only in the very periphery but also in deeper areas of the cortex.

### Substantial presence of pluripotency factor mRNAs in the neonatal ovary

**Figure 2:**
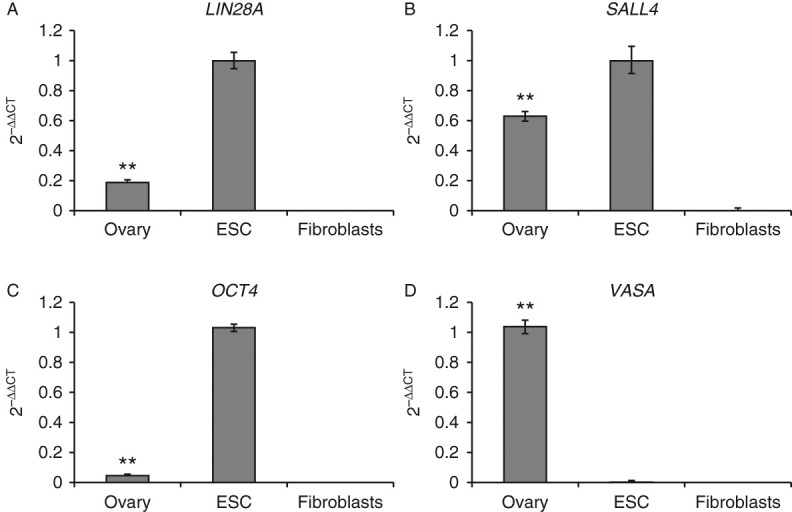
mRNA expression of pluripotency and germ cell markers in the neonatal marmoset ovary compared with pluripotent ES cells and fibroblasts. ES cells serve as positive controls for pluripotency markers and fibroblasts as negative controls. The value for ovary (*VASA*) or for ES cells (*OCT4A*, *SALL4*, and *LIN28A*) was always set at 1. **P<0.01 between ES cells and ovary. For more information, see results.

To corroborate and confirm the expression of LIN28A on the mRNA level, we performed RT-qPCR for *LIN28A* (Fig. 2A). Marmoset monkey ES cells and fibroblasts were used as positive and negative controls respectively. In fibroblasts, *LIN28A* mRNA was undetectable. By contrast, neonatal ovary exhibited robust *LIN28A* transcript levels. We further tested the expression of the germ-line- and pluripotency-associated factors *SALL4* and *OCT4A*. In fibroblasts, *SALL4* mRNA was only very weakly expressed, while *OCT4A* was undetectable. For *SALL4*, the ovary showed strong expression (Fig. 2B). *OCT4A* was also clearly detectable (Fig. 2C). As an additional control, we tested the expression of the germ cell gene *VASA* (*DDX4*). *VASA* transcripts were highly abundant in neonatal ovary, while only very low *VASA* transcript levels were detected in undifferentiated ES cells and fibroblasts (Fig. 2D). As we compared the expression of genes in pure cell populations (ES cells and fibroblasts) with their expression in a tissue containing several cell types (ovary), these data cannot be directly related to a cell-specific expression level in the ovary. However, very importantly, the signals detected in ovary were always significantly above the background levels detected in fibroblasts (*P*<0.01). In summary, Fig. 2 clearly shows that the neonatal marmoset monkey ovary contains substantial amounts of transcripts not only of *VASA*, but also of pluripotency markers.

### Pre- and neonatal ovarian germ cells express pluripotency factors

**Figure 3:**
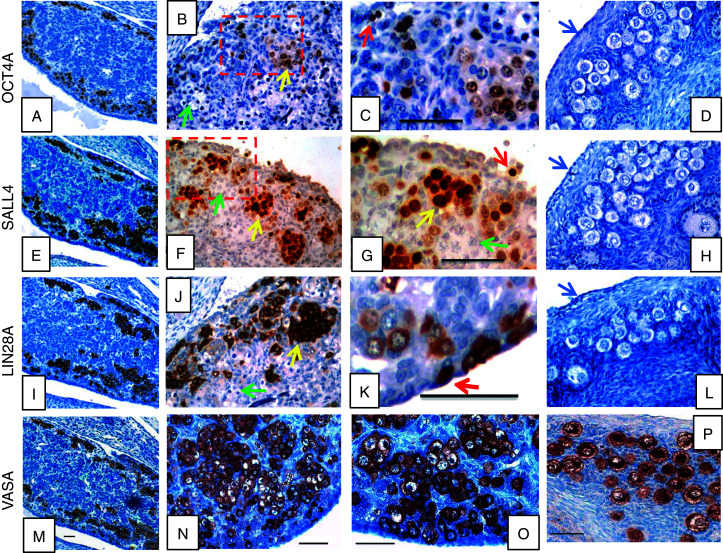
Immunohistochemical analysis of OCT4A, SALL4, LIN28A, and VASA protein expression in fetal, neonatal, and adolescent marmoset monkey ovaries. OCT4A was detected in early fetal PGCs and oogonia (A). In the neonatal ovary, OCT4A was present in premeiotic germ cells (B and C). Note the small structures strongly stained for OCT4A in C (red arrow). OCT4A was neither observed in germ cells nor in the ovarian surface epithelium (blue arrow) of the 1-year-old ovary. (E) Strong SALL4 staining of germ cells in the fetal gonad. In the neonatal gonad, premeiotic germ cells were strongly stained. There were also cells of the OSE, which are stained (red arrow in G), that had a significantly smaller nucleus than typical oogonia (F and G, yellow arrows). Meiotic cells exhibited only faint SALL4 signals (green arrows). In older stages, SALL4 was almost undetectable (H). (I) LIN28A was also specifically expressed in virtually all germ cells of the fetal gonad. In the neonatal gonad, LIN28A was found in oogonia and they were intermingled with or part of the OSE in some cells (J and K). No LIN28A-positive cells were observed in the older ovary (L). VASA, as a general germ cell marker, was present in all germ cell stages (M, N, O, and P). The scale bars represent 50 μm. In cases where only one scale bar per column is present, it applies to all pictures in the respective column.

In order to analyze the cell-specific distribution of selected pluripotency markers in the neonatal marmoset ovary, we performed immunohistochemistry for OCT4A, SALL4, and LIN28A. Additionally, we stained for the general germ cell marker VASA. Staining results of the neonatal ovarian samples are shown in Fig. 3. As references, we included one fetal (Fig. 3A, E, I and M), one 1-year-old (Fig. 3D, H, L and P), and three adult marmoset ovaries (data not shown). The early fetal ovary contained germ cells in the developing ovarian cortex. The germ cells were mainly present in clusters and expressed OCT4A (Fig. 3A). Based on morphology, we found no germ cell in the fetal ovary lacking OCT4A signals. When we tested for SALL4, we again found all germ cells of the fetal ovary expressing this transcription factor (Fig. 3E). The same was found for LIN28A (Fig. 3I) and VASA (Fig. 3M). Hence, all three pluripotency factors, OCT4A, SALL4, and LIN28A, were robustly expressed by oogonia in the fetal ovary.

In the neonatal ovary, many of the germ cells with a nuclear chromatin structure indicating a premeiotic stage were OCT4A positive (Fig. 3B). Those germ cells that entered meiosis lacked OCT4A signals. Somatic cells were also OCT4A negative. We also detected some structures significantly smaller than regular germ cell nuclei that were strongly positive for OCT4A (Fig. 3C, red arrow). The 1-year-old marmoset ovary (Fig. 3D) and the adult ovaries (not shown) lacked detectable OCT4A. The SALL4 expression pattern also showed very intense signals for premeiotic germ cells in the neonatal ovary (Fig. 3F and G). In contrast to OCT4A, weak SALL4 staining was also observed in oocytes in meiotic prophase. Most cells of the OSE were negative for SALL4. However, some of the OSE cells were very intensely stained for SALL4 (Fig. 3G). In the 1-year-old marmoset ovary, SALL4 was basically undetectable (Fig. 3H). Applying LIN28A immunohistochemistry, only premeiotic germ cells were labeled. Most of them were still organized in clusters, but some were also present as isolated germ cells (Fig. 3J). At some sites, the OSE contained several cells being strongly LIN28A positive, while the neighboring cells of the OSE were LIN28A negative (Fig. 3K). Older postnatal stages were found to be LIN28A negative (Fig. 3L). VASA was detectable in premeiotic as well as meiotic germ cells (Fig. 3M, N, O and P). In summary of this part, the neonatal marmoset ovary contains numerous premeiotic germ cells expressing OCT4A, SALL4, and LIN28A. Some OSE cells were also positive for SALL4 and LIN28A. However, no OCT4A-positive cells were found in the OSE.

**Figure 4:**
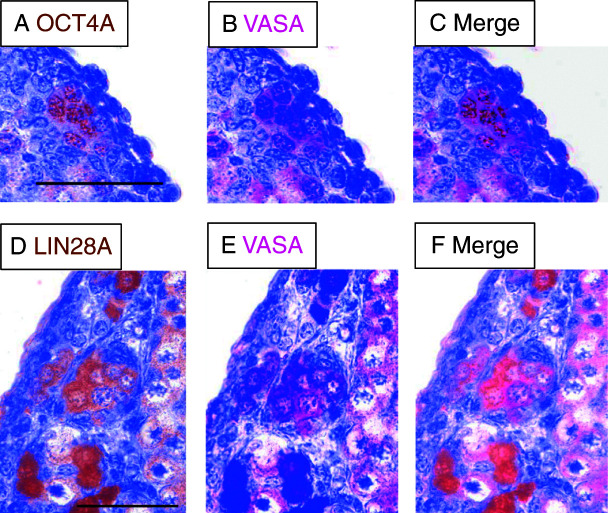
Co-localization of pluripotency markers with the general germ cell marker VASA in neonatal marmoset ovary. (A) OCT4A-stained oogonia. The OCT4A signals are nuclear. (B) The same individual tissue section shown in A stained for VASA, which is predominantly cytoplasmic. (C) Merged stainings from A and B indicate that individual cells express both OCT4A and VASA. (D) LIN28A-stained oogonia. (E) The same tissue section shown in D stained for VASA. (F) Merged stainings from D and E. The scale bars represent 50 μm.

In order to further corroborate the germ cell identity of the pluripotency factor-positive cells, we performed immunohistochemical double-staining procedures for OCT4A/VASA and LIN28A/VASA (Fig. 4). Cells expressing nuclear OCT4A also had the VASA-positive cytoplasm (Fig. 4A, B and C). In premeiotic cells, there was also an almost complete overlap between LIN28A and VASA (Fig. 4D, E and F). Only those cells with a nuclear morphology indicating entry into meiosis had low or no LIN28A expression, but were positive for VASA.

### Premeiotic germ cells express the proliferation marker MKI67

**Figure 5:**
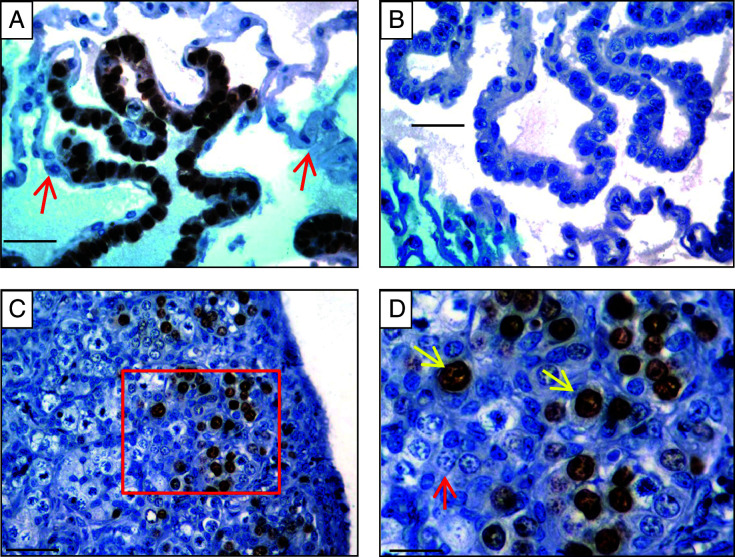
Expression analysis of the proliferation marker MKI67 in the neonatal marmoset ovary. Marmoset ES cells as highly proliferative cells were used as positive controls for the Ki-67 antibody (A, brown staining). Proliferation-arrested mouse embryonic feeder cells were not stained (red arrows). (B) Replacement of the primary antibody by the respective rabbit IgG produced no signals. (C) In neonatal marmoset ovaries, the nuclei of the oogonia in the outer zone of the cortex were strongly and specifically labeled (D, yellow arrows). Neither the cytoplasm of oogonia nor oocytes (red arrow) were stained. The scale bars represent 50 μm.

We were interested to know whether the premeiotic germ cells in the neonatal ovary express the proliferation marker MKI67. We used marmoset monkey ES cells as a positive control for highly proliferative marmoset cells (Muller *et al*. 2009). Figure 5A shows intense nuclear signals for MKI67 in marmoset monkey ES cells indicating cross-reactivity of the antibody with the marmoset Ki-67 antigen. By contrast, proliferation-arrested mouse feeder cells were not stained. Replacement of the primary antibody with non-specific rabbit IgGs did not produce any signals (Fig. 5B). When we stained neonatal marmoset ovaries for MKI67, many germ cells in the outer cortical zone were positive (Fig. 5C). This was also confirmed (data not shown) using a second independent Ki-67 antibody (Dako M7240; clone MB-1), which was used in previous studies with marmosets (McKinnell *et al*. 2013). A higher magnification (Fig. 5D) revealed that the stained cells were predominantly premeiotic germ cells, while germ cells that entered meiosis were not stained. Only few putative somatic cells in the outer cortical zone were stained. Meiotic germ cells were MKI67 negative. These data show that marmoset monkey neonatal ovaries contain a significant population of proliferating premeiotic germ cells.

### Co-localization of MKI67 with pluripotency markers OCT4A and LIN28A

**Figure 6:**
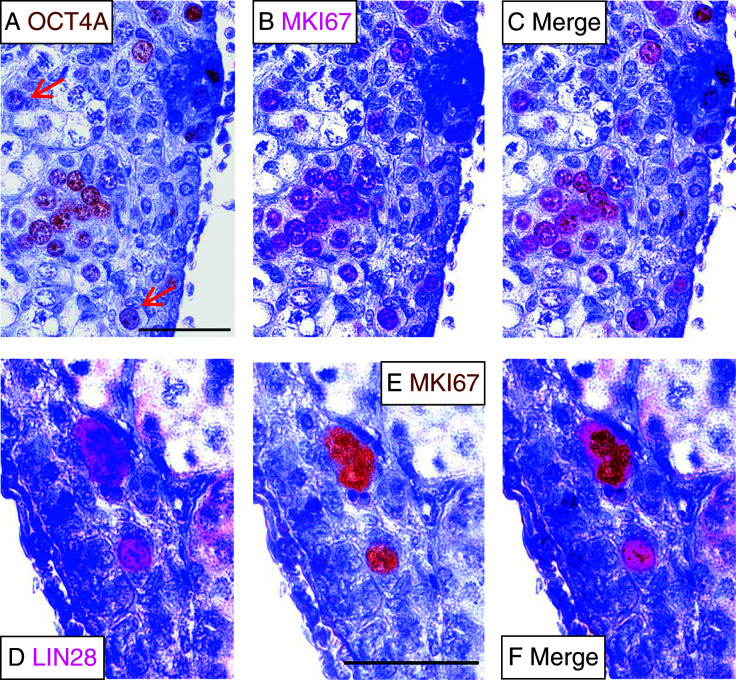
Co-localization of pluripotency markers with the proliferation marker MKI67 in neonatal marmoset ovary. (A) OCT4A-stained oogonia. Oogonia with weak OCT4A signals are highlighted by a red arrow. (B) The same tissue section shown in A stained for MKI67. (C) Merged stainings from A and B. (D) LIN28A-stained oogonia. (E) The same tissue section shown in D stained for MKI67. (F) Merged stainings from D and E.

Finally, we were interested to know whether individual cells express both pluripotency markers and the proliferation marker MKI67. Therefore, we tested the combinations OCT4A/MKI67 and LIN28A/MKI67 in double-staining procedures. Figure 6A, B and C shows that the co-localization of OCT4A with MKI67 is almost complete in oogonia organized in cell nests. By contrast, individual oogonia still positive for MKI67 exhibit only weak OCT4A signals (arrows in Fig. 6A). Co-staining for LIN28A and MKI67 revealed that MKI67-positive cells were also LIN28A positive (even individual cells outside of cell nests) (Fig. 6D, E and F).

## Discussion

In this study, we demonstrated that the neonatal marmoset ovary exhibits a histologically undifferentiated zone underneath the OSE. This zone is termed the ICZ. This zone has no counterpart in the neonatal human ovary (van Wagenen & Simpson 1973, Sforza *et al*. 1993). In contrast to the human neonatal ovary, the marmoset ovary did not exhibit surface irregularities or deep organ lobulation (van Wagenen & Simpson 1973). The neonatal marmoset monkey ovary also contains numerous premeiotic germ cells. This is clearly in contrast to the human ovary, where the vast majority of oogonia enter meiosis early during fetal development (Stoop *et al*. 2005, Hartshorne *et al*. 2009). In the postnatal human ovary, there are only very few premeiotic germ cells at birth (Byskov *et al*. 2011). This striking difference between humans and the marmoset makes the marmoset very interesting with regard to at least two aspects: the first one is that ‘early’ primate ovary development can be experimentally studied in postnatal animals. Hence, using the marmoset, it is possible to test the action of hormones or other bioactive substances on the progression of oogonia to meiosis and on folliculogenesis *in vivo*. This might be of special relevance in the area of reproductive toxicology. The second aspect is the relatively easy retrieval of proliferating primitive germ cells in order to try to establish germ-line-derived pluripotent cell lines from the marmoset. Indeed, pluripotent cells were previously derived from human primordial germ cells (PGCs) (Shamblott *et al*. 1998, 2001). PGCs express many pluripotency markers such as OCT4 (previous studies did not discriminate between OCT4 and OCT4A) or LIN28A. As the postnatal marmoset oogonia appear to have a similar marker profile as PGCs, it is conceivable that the MKI67-positive marmoset oogonia can be cultured and expanded under appropriate conditions. Besides ES cells and iPS cells, such germ-line-derived pluripotent cells might be an additional option to obtain pluripotent primate cells for comparative molecular studies on pluripotent cells of different origin and for testing the general suitability of germ-line-derived pluripotent stem cells in regenerative medicine.

The developing human (Hartshorne *et al*. 2009, Byskov *et al*. 2011) and rhesus macaque (van Wagenen & Simpson 1973) ovaries have been studied in detail. Both are representatives of the old world primates (Catarrhini). Herein, we studied the developing marmoset monkey ovary as a representative of the new world primates (Platyrrhini) and found clear differences between these groups with regard to the presence of premeiotic germ cells in neonatal ovaries. Both the new world and old world primates belong to the simians (Anthropoidea). Despite the differences in the neonatal ovary, the Anthropoidea tested so far share the characteristic of absence of premeiotic germ cells in the adult ovary. Interestingly, in the primate group of prosimians (Strepsirrhini), even in the adult ovary, proliferating premeiotic germ cells are present, as very solid histological and isotope incorporation studies have already shown some decades ago (Ioannou 1967, Butler & Juma 1970). Unfortunately, the access to gonads of Strepsirrhini is extremely limited or practically almost impossible today. However, it would be of major interest to extend the more detailed investigations on ovarian and germ cell development, which are possible with today's tools, to Strepsirrhini. Availability of Strepsirrhini would probably offer the opportunity to study female primate germ cell proliferation and folliculogenesis over the lifetime of the animal and not only during a short period of intrauterine or postnatal development. Furthermore, it should be taken into consideration that human ovarian development with very early loss of the premeiotic germ cell population might be an extreme peculiarity within the whole group of primates.

The differentiation states of the somatic cell populations in the neonatal ovary were not the objective of this study, which focused on premeiotic germ cells. However, the general histological immaturity of the neonatal marmoset gonad, especially the presence of the ICZ, suggests that the somatic cells might also be in a very primitive, supposedly fetal, state when compared with human ovarian development. Although a detailed analysis is not provided in this study, it is important to note that the ICZ of the neonatal marmoset monkey ovary is different from the primitive cortical tissue (PCT) that has been described for the human fetal and neonatal ovary (Sforza *et al*. 1993). Hence, the ICZ of the neonatal marmoset ovary has no direct histological counterpart in the neonatal human ovary (van Wagenen & Simpson 1973, Forabosco *et al*. 1991, Sforza *et al*. 1993). The kind of description of the PCT by Sforza *et al*. (1993) strongly suggests that even the 20-week-old human fetal ovary is more mature than the neonatal marmoset ovary.

Using an antibody against activated caspase 3, which is a specific marker of apoptotic cells, we were able to detect numerous positive cells in the neonatal thymus (positive control) and in a neonatal ovary that was cultured for 6 h before fixation (Supplementary Figure 1, see section on supplementary data given at the end of this article). The freshly fixed neonatal ovary exhibited relatively few activated caspase 3-positive cells, i.e. apoptotic germ cells, as has been reported for the fetal human ovary (Fulton *et al*. 2005). However, due to the long period of time during which the oogonial population is present during development, even the presence of only few apoptotic germ cells at a specific time point may have considerable consequences for the whole germ cell population over time (Fulton *et al*. 2005). This has to be studied in the marmoset ovary in more detail in the future. Nevertheless, the small structures strongly stained for OCT4A in Fig. 3C may represent a degenerating nucleus of an apoptotic oogonium.

In general, marmoset monkey embryos exhibit a developmental delay during the first half of gestation, which has been known for a long time (Phillips 1976, Merker *et al*. 1988). However, the (molecular) mechanisms inducing this developmental delay are not understood. Regarding the delayed gonad development, however, it is very likely that the regular birth of co-twins, which are frequently of different sexes, favors delayed gonadal development. In humans, it is well known that phases of high sex steroid production occur already during fetal development (Scott *et al*. 2009). In case of twin fetuses of different sexes, such a high fetal sex steroid production may interfere with the proper development of the other sex. This is a well-described phenomenon, for instance, in cattle, where infertile freemartins are a rather common phenomenon (Padula 2005). Therefore, it is plausible that endocrine-controlled phases of gonad development are postponed to postnatal periods in marmosets.

In conclusion, the neonatal marmoset monkey ovary is very immature compared with the neonatal human ovary. It contains a novel histological layer called the ICZ of the neonatal marmoset ovary. The ICZ has no counterpart in the developing human ovary. The neonatal marmoset ovary also contains numerous oogonia positive for pluripotency factors and the proliferation marker MKI67, which is clearly in contrast to the human neonatal ovary. Our findings may allow deeper investigations of the transition of primate oogonia to oocytes and of folliculogenesis in NHPs *in vivo*.

## Supplementary data

This is linked to the online version of the paper at http://dx.doi.org/10.1530/REP-14-0068.

Supplementary Figure

## Figures and Tables

**Table 1:** Primer sequences, sizes of PCR products, and concentrations of primers.

**Primers**	**Primer sequence**	**PCR product size** (bp)	**Concentration** (nM)
Cj_*GAPDH*_Fw	5′-TGCTGGCGCTGAGTATGTG-3′	64	300
Cj_*GAPDH*_Re	5′-AGCCCCAGCCTTCTCCAT-3′		50
Cj_*LIN28A*_Fw	5′-GACGTCTTTGTGCACCAGAGTAA-3′	67	300
Cj_*LIN28A*_Re	5′-CGGCCTCACCTTCCTTCAA-3′		50
Cj_*SALL4*_Fw	5′-AAGGCAACTTGAAGGTTCACTACA-3′	77	900
Cj_*SALL4*_Re	5′-GATGGCCAGCTTCCTTCCA-3′		50
Cj_*VASA*_Fw	5′-TGGACATGATGCACCACCAGCA-3′	210	50
Cj_*VASA*_Re	5′-TGGGCCAAAATTGGCAGGAGAAA-3′		900
Cj_*OCT4A*_Fw	5′-GGAACAAAACACGGAGGAGTC-3′	234	300
Cj_*OCT4*_Re	5′-CAGGGTGATCCTCTTCTGCTTC-3′		50
