# Supplementary material for: The neonatal marmoset monkey ovary is very primitive exhibiting many oogonia
Source: Reproduction. 2014 Mar 24;148(2):237–47. doi: 10.1530/REP-14-0068 (PMC4086814; doi:10.1530/REP-14-0068)
Supplement: Supplementary Figure [file supp_REP-14-0068_Supplementary_figure_1.pdf]

Fereydouni et al. supplementary figure 1

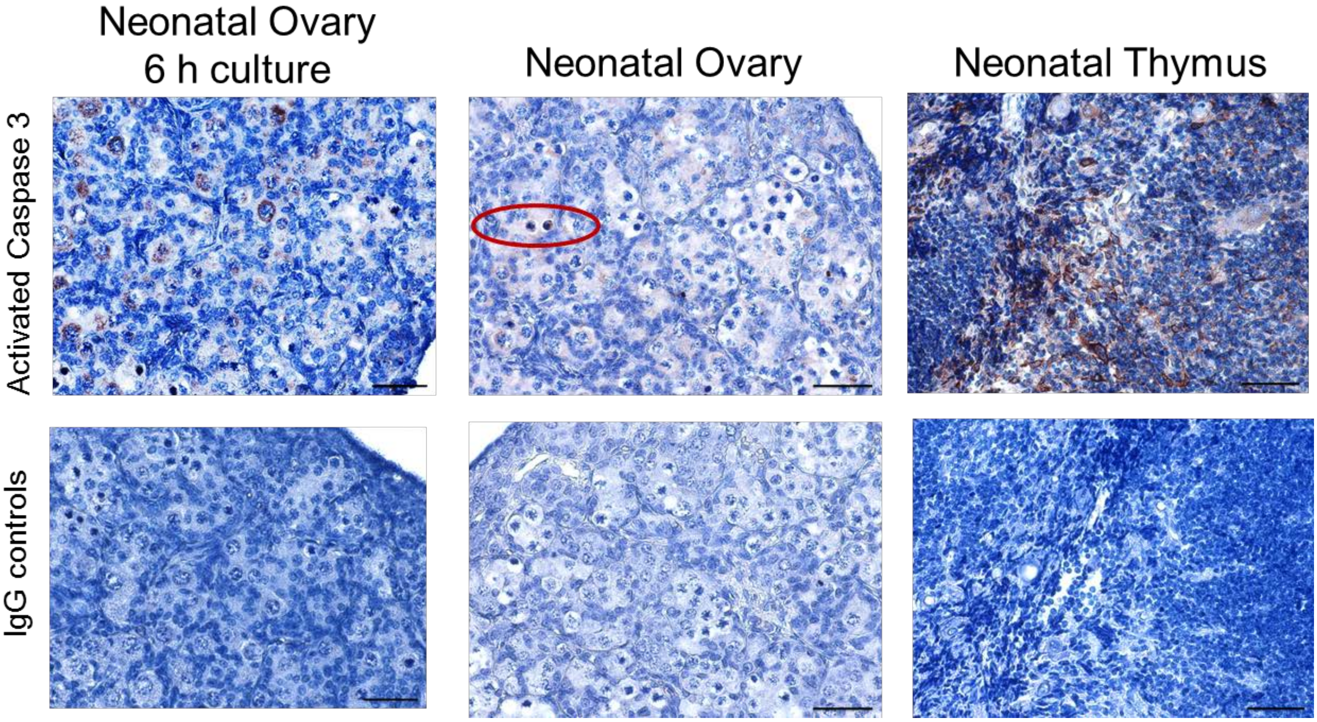

Supp. Fig. 1: Caspase 3 detection indicating apoptosis in neonatal marmoset monkey ovary. Using an antibody against activated Caspase 3, which is a specific marker of apoptotic cells, numerous positive cells were detected in the neonatal thymus (positive control, right panel) and in a neonatal ovary which was cultured for 6 hours before fixation (left panel). In contrast, the freshly fixed neonatal ovary exhibited only very few activated caspase 3-positive germ cells suggesting that apoptosis is not a major mechanism to regulate the germ cell population in the neonatal marmoset ovary.

Caspase 3 antibody (#ab2302, Abcam) was used at a 1:50 dilution to detect activated Caspase 3. The staining procedure was similar to the described in the main text with the exception that the antigen retrieval step was replaced by a Proteinase K (50µg / ml 0.05 M Tris-buffered saline) digestion of the sections for 15 minutes at room temperature.

IgG controls did not show any signals.
